# In Vivo Characterisation of Skin Response to Sustainable Car Cleaning Products

**DOI:** 10.3390/ma19020269

**Published:** 2026-01-09

**Authors:** Bartosz Woźniak, Marta Marzec, Agata Wawrzyńczak, Izabela Nowak

**Affiliations:** Department of Applied Chemistry, Faculty of Chemistry, Adam Mickiewicz University in Poznań, Uniwersytetu Poznańskiego 8, 61-614 Poznań, Poland; barwoz6@amu.edu.pl (B.W.); marta.marzec@amu.edu.pl (M.M.)

**Keywords:** car cosmetics, bio-based products, application tests, in vivo testing, skin barrier, skin compatibility

## Abstract

Synthetic surfactants are currently the most commonly used agents in human cosmetics and household chemicals. However, there are increasingly frequent reports of cases showing the negative impact of these surfactants on human skin. Out of concern for users, many companies, including those originating in the automotive chemicals industry, are increasingly turning to surfactants that are more dermatologically friendly and non-toxic to the environment. The following study aimed to examine two custom-developed car shampoo concentrates based on highly biodegradable raw materials and to analyse their impact on selected skin parameters. The research included semi-contact patch tests and in vivo instrumental tests on a group of volunteers, measuring the following parameters: skin moisturising, transepidermal water loss (TEWL), pH, roughness, smoothness, and skin scaliness. Both products showed very good dermatological tolerance, without causing drastic or long-lasting changes in selected skin parameters. The results of the tests confirmed that both car products can represent a safe alternative for everyday use, in accordance with the principles of green chemistry.

## 1. Introduction

One of the fastest-growing sectors of the chemical industry is the surfactant industry. This well-known group of chemical compounds is widely used in many areas of production, including detergents, cosmetics, pharmaceuticals, paints, and car care products [1,2,3,4,5,6,7,8]. Their universal application is primarily due to their ability to reduce surface tension, their excellent cleaning properties, and their high solubility in water. However, in recent years, there has been a growing interest in the impact of synthetic surfactants on the environment and human health [3,9,10,11]. This phenomenon is linked to increased environmental awareness among consumers and growing market pressure to use safe, biodegradable raw materials. An increasing number of scientific reports point to the adverse effects of synthetic surfactants on the skin, including potential irritation, disruption of the lipid barrier, and even inflammatory reactions [1,10]. Even in low concentrations, they can disrupt the functions of the epidermal barrier and affect the structural proteins of the skin, which poses a significant challenge for the cosmetics and household chemicals industries. As a result, biosurfactants, i.e., natural surface-active compounds that are an alternative to synthetic surfactants, are becoming increasingly important. They are characterised by high biodegradability, low toxicity, good resistance to changing pH and temperature conditions, and no harmful impact on the environment. The growing popularity of biosurfactants is due to their compliance with the principles of green chemistry and sustainable development [3,8,12].

It is common practice in the formulation of detergent and cosmetic products to use mixtures of surfactants with different physicochemical properties. This approach allows for a synergistic effect, reducing the potential toxicity of the product and the risk of irritation to the skin and mucous membranes, of the eyes [1,13,14,15]. One group with great potential for use in cleaning products is based on alkyl polyglycosides (APGs). This is a group of non-ionic surfactants of plant origin, obtained mainly from sugars and vegetable oils. APGs are characterised primarily by a very favourable dermatological profile, low toxicity, and high skin compatibility. Thanks to their natural origin and high biodegradability, alkyl polyglycosides are in line with the principles of green chemistry and the concept of sustainable development. Environmentally friendly biomass-based surfactants like alkyl polyglycoside (APG) and tea saponin (TAS) were used as foaming agents in obtaining detergents enabling effective cleaning of different surfaces, including stainless steel and glass [16]. Compared to commonly used synthetic surfactants, APGs (especially raw materials like coco-glucoside; decyl glucoside) have a lower irritation potential, which makes them particularly useful in products intended for people with sensitive skin [14,17,18,19,20].

Another important group of non-ionic surfactants widely used in the detergent industry involves fatty alcohol ethoxylates (FAEs), mostly C12-C15 fatty alcohol ethoxylates. They are mainly characterised by very good cleaning properties, high biodegradability, low toxicity, and resistance to water hardness. For example, FAEs based on C12–C14–EO30 and C16–C18–EO30 systems were successfully applied as corrosion inhibitors of mild steel [21]. In addition, FAEs are highly compatible with other types of surfactants, and their relatively low production costs make them an attractive alternative to other biodegradable surfactants such as APGs [1,14,22,23,24].

The skin, which is the largest organ in the human body, acts as a natural protective barrier against external factors. Especially, the stratum corneum plays a key role in maintaining its integrity. It is composed of corneocytes embedded in a lipid matrix. Lipids such as ceramides, cholesterol, and free fatty acids form the so-called hydrolipid barrier, which limits transepidermal water loss (TEWL) and ensures proper skin hydration. Natural moisturising factors (NMF), including amino acids, lactates, and urea, help maintain optimal moisture levels. Slightly acidic pH of the skin, ranging from 4.5 to 5.5, promotes the proper development of microflora and the activity of enzymes involved in epidermal renewal. Disruption of the hydrolipid barrier function, e.g., as a result of using detergents, leads to an increase in pH, but also to amplified TEWL, reduced moisture, and the occurrence of irritation, dryness, or inflammation of the skin. Severe disruption of this barrier may even promote the development of allergic and autoimmune diseases. Therefore, the appropriate selection of raw materials for cleansing products, including the type of surfactants used, is crucial for user safety [25,26,27].

Despite numerous studies on the impact of cosmetics and detergents on skin parameters, there is still a lack of data in the literature analysing the analogous impact of car care products. These products, often used directly by consumers, may cause long-term exposure of the skin to surfactants and other chemical ingredients. This study aimed to conduct a dermatological evaluation of two custom-developed car shampoos and to analyse their impact on selected skin parameters using instrumental testing. The products were developed based on selected surfactants that combine very good cleaning properties with limited irritant potential. The study included semi-contact patch tests and application tests on a group of volunteers. The results were supplemented with subjective assessments by the participants and statistical analysis.

## 2. Materials and Methods

### 2.1. Characterisation of Car Shampoos

In previous studies, seven original formulas of car shampoo concentrate were developed, of which two products with the best cleaning and foaming properties were selected based on application tests [14]. The developed products consisted only of raw materials with a very high level of biodegradability, confirmed by OECD 301 standards [28] for each of the raw materials. Both car shampoos were prepared in the laboratory of the Department of Applied Chemistry, Faculty of Chemistry, Adam Mickiewicz University, Poznań, Poland. The developed products were packaged in appropriately labelled bottles with applicators attached, which allowed for their easy identification and uniform application among all participants. The list of raw materials used for both shampoos is presented below.

Car Shampoo 1

Ingredients: Aqua, C8-10 Alcohol Ethoxylate (2 EO), Caprylyl/Capryl Glucoside (40–44%), Cocamidopropyl Betaine, 3-Methoxy-3-methyl-1-butanol, Glycerin, Trisodium Dicarboxymethyl Alaninate, Phenoxyethanol, Polymer dye (55965-84-9), Parfum.

Car Shampoo 2

Ingredients: Aqua, C8-10 Alcohol Ethoxylate (2 EO), Caprylyl/Capryl Glucoside (58–62%), Cocamidopropyl Betaine, 3-Methoxy-3-methyl-1-butanol, Glycerin, Trisodium Dicarboxymethyl Alaninate, Phenoxyethanol, C.I. Acid Violet 126, Parfum.

### 2.2. Dermatological Tests

Patch tests were conducted to assess the local skin tolerance of the product by applying a single patch test and reading the skin reaction after 48 h and 72 h, and in the case of positive skin reactions, also after 96 h. Twenty healthy women were recruited for the study, 14 of whom had a positive allergy test result. The group of participants included 9 with dry skin, 8 with normal skin, and 3 with combination skin. The age range of the participants was 26 to 65 years. The test product in the form of a 1 wt.% aqueous solution (0.1 mL) was applied to filter paper discs, which were attached with a hypoallergenic plaster to the skin of the arm on the extensor side or on the back. The samples were removed after 48 h. The first skin reaction reading was taken 15 min after removing the sample, and the next one after 72 h. Skin reactions were assessed according to a scale commonly used in dermatological studies. The total duration of the study was three days.

### 2.3. Application and Instrumental Tests

The in vivo studies were approved by the Bioethics Committee of the Medical University of Poznań (328/25, 8 May 2025). The study was short-term, reaching 2.5 h per participant. All participants were informed about the details of the study, signed a consent form to participate in the study, and received information materials about the tested products. Each participant answered all the questions in the questionnaire, which authorised them to participate in the study. After a minimum of 15 min of acclimatisation in the room, each participant underwent preliminary measurements to obtain zero values. Then, 1 mL of the tested product was applied to each of the inner sides of the participants’ forearms: shampoo 1 to the left forearm and shampoo 2 to the right forearm. The participants were asked to wash each forearm with the appropriate shampoo and then rinse the product thoroughly with water. Subsequent instrumental measurements were taken 15, 60, and 120 min after the application of the shampoos. Each of the tested products was used in the form of a 3 wt.% aqueous solution.

#### 2.3.1. Characteristics of the Group

The test group consisted of 20 adult volunteers, including 15 women and 5 men. The participants ranged in age from under 25 to over 55. They assessed the condition of their skin based on a self-evaluation questionnaire. Among the volunteers, 14 reported having normal skin, 1 had dry skin, 3 had skin prone to irritation, 1 had combination skin prone to irritation, and 1 had allergic skin prone to irritation. Each participant received two car shampoo solutions to test, which were applied once to the inner sides of their forearms, in accordance with the instructions provided in advance. In addition, the participants were advised not to use any products that could significantly affect the results and to avoid intense physical activity during the study period.

#### 2.3.2. Equipment for Analysing Skin Parameters

The analysis of the biophysical parameters of the skin was carried out using devices from Courage and Khazaka Electronic GmbH (Cologne, Germany). This equipment enabled an accurate and detailed assessment of the impact of the tested products on the condition of the skin barrier. The tests were painless and non-invasive. The testing process involved applying a measuring probe to a selected area of skin on the forearm and recording the data transmitted from the probe to the measuring system on a computer. The following equipment was used for the tests:

Corneometer CM825—used to determine the moisture level of the skin surface (stratum corneum). The device enables very fast measurement (measurement time: 1 s), which allows the occlusion effect to be avoided. The principle of operation is based on the difference in electrical conductivity between dry and moist skin. Water has a high capacity to store electrical charge, while other substances have a much lower capacity. The higher the water content in the stratum corneum, the higher the dielectric constant and the higher the measurement result [29,30,31]. Five measurements were taken for each participant, from which the average value for each forearm was calculated. The measurements were taken on both forearms at time points t0, t15, t60, and t120 by applying the device to the tested skin area.

Tewameter TM 300—a measuring device used to determine transepidermal water loss (TEWL). Water evaporation from the skin surface is a natural physiological process, but when the epidermal barrier function is weakened, the TEWL value increases significantly. The device indirectly measures the water vapour density gradient above the skin surface, using two pairs of sensors placed inside an empty measuring cylinder. The measurement is carried out in an open chamber, so it does not disturb the skin’s microenvironment. The measurement process involves applying the probe to the skin surface so that the short end of the head is in contact with the skin with the appropriate pressure [29,31,32,33,34,35]. As a result of the tests, an average value was obtained from 20 measurements taken for each of the two shampoos tested at four time points (t0, t15, t60, and t120).

Visioscan VC98—enables in vivo analysis of skin surface topography, including assessment of key parameters such as smoothness (SEsm), roughness (SEr), and scaliness (SEsc). The measurement involves recording an image of the examined skin area using a CCD camera, whose signal is transmitted to a computer for further analysis. The device consists of a video sensor, a lens, and a built-in UVA light source, which allows for high-quality imaging of a 6 × 8 mm area of skin [29,36,37]. During each test, three photographs of the skin of each forearm were taken, covering the previously designated measurement areas. The results were obtained for each of the two shampoos tested at four time points (t0, t15, t60, and t120).

Skin-pH-meter PH 905—a device for quick and non-invasive in vivo measurement of skin surface pH. The measurement involves placing the probe head in close contact with the skin surface of the tested area [29,38,39]. A series of five measurements was taken for each participant and then averaged. The final results were obtained for both tested products at four time points (t0, t15, t60, and t120).

### 2.4. Statistical Analysis

The data obtained from the application tests at four time points (t0, t15, t60, and t120) were subjected to statistical analysis using Statistica 13.3 (StatSoft, Krakow, Poland). In order to confirm that the statistical test assumptions were met (separately for each analysed group), Levene’s test was performed to assess the homogeneity of variance. The correctness of the parametric test selection was related to the lack of statistical significance of this test (*p* > 0.05). The statistical significance of changes in skin parameters was assessed using the non-parametric Kruskal–Wallis test (for TEWL, SEr, SEsm, and SEsc parameters) and one-way ANOVA (for other parameters). In the analyses, the dependent variable was the numerical value of a given skin parameter, the qualitative variable was the measurement time, and the random factor was the volunteers. For parameters with statistically significant differences, appropriate post hoc tests were performed: Tukey’s test for different N (in the case of one-way ANOVA) and Dunn’s test (for the Kruskal–Wallis test). A *p*-value of <0.05 was considered statistically significant.

## 3. Results

### 3.1. Dermatological Study—The Patch Test

None of the 20 study participants, including 14 individuals with a history of allergies, showed positive reactions either 48 h or 72 h after application of the solutions of car shampoo formulations (Table S1). None of the participants experienced any positive skin reactions. Therefore, the study was not continued for 96 h after application. These results indicate that the developed products do not have irritating or sensitising properties. Semi-open contact tests confirm that our custom-developed formulations meet the requirements of the skin compatibility test.

### 3.2. In Vivo Application Studies

This section presents the results of research on the impact of custom-developed car shampoos on selected skin parameters. The research was conducted to assess the impact of the ingredients used in the developed formulations on the condition of the skin of people who come into direct contact with them. The analysis covered changes in skin hydration, transepidermal water loss (TEWL), pH, and skin surface topography, including roughness (SEr), scaliness (SEsc), and smoothness (SEsm). The results obtained were also subjected to statistical analysis.

#### 3.2.1. Effect on Skin Hydration

**Figure 1:**
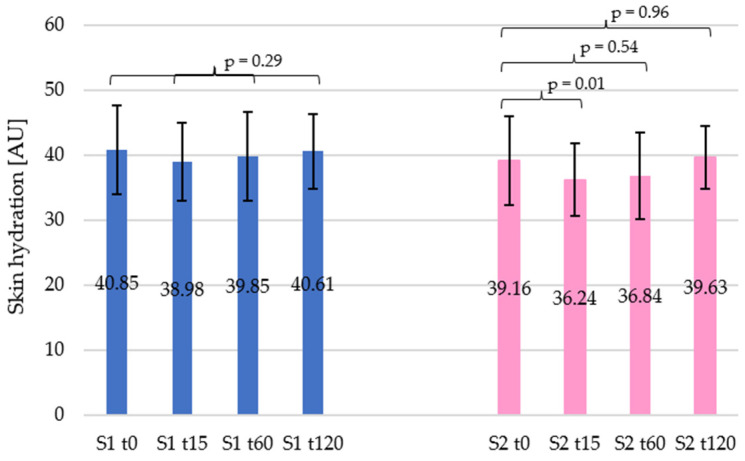
Changes in skin hydration over time for the car shampoos tested. Blue and pink bars represent car shampoo 1 (S1) and car shampoo 2 (S2), respectively.

For both studied car shampoo samples, skin hydration underwent similar changes (Figure 1). In the initial stages, a decrease in the parameter studied was observed, which then gradually returned to its original state. For shampoo 1, this value decreased by 4.58% at t15, by 2.45% at t60, and only by 0.60% at t120, compared to the initial value. However, statistical analysis did not show these changes to be significant (*p* = 0.29). In contrast, shampoo 2 showed a greater decrease in the moisture value at t15, namely by 7.50% (*p* = 0.01), which changed to 5.94% at t60 (*p* = 0.54) and to 1.41% at t120 (*p* = 0.96) relative to the initial value. Therefore, the only statistically significant change was a decrease in moisture after 15 min for shampoo 2.

In summary, both shampoos caused an initial decrease in skin hydration (most noticeable after 15 min), which then gradually returned to baseline levels, as shown in Figure 1. This observation is consistent with the results of a one-way ANOVA significance test, indicating no statistically significant changes in moisture levels at most time points, which is a desirable phenomenon in the context of this study.

#### 3.2.2. Effect on Transepidermal Water Loss

**Figure 2:**
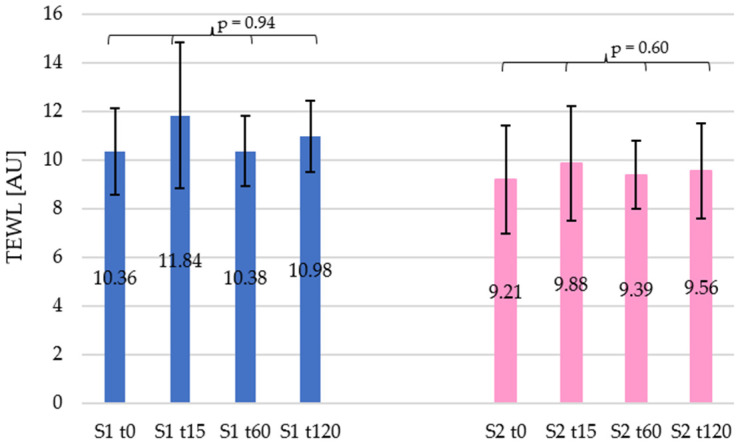
Changes in transepidermal water loss (TEWL) over time for the car shampoos tested. Blue and pink bars represent car shampoo 1 (S1) and car shampoo 2 (S2), respectively.

In the case of transepidermal water loss (TEWL) through the skin, a change for both tested shampoos was observed, especially in the initial stages of measurement (Figure 2). For shampoo 1, at time t15, the TEWL value increased by 14.34%. Subsequent measurements showed a decrease compared to the t15 measurement, as at t60 the total TEWL increase was only 0.23%, compared to the initial value, which then rose to 6.04% at t120. Shampoo 2 had a significantly lower effect on TEWL. At t15, the increase was 7.27%, whereas at t60 and t120 it was 1.87% and 3.78%, respectively, compared to the initial value. This shampoo had a smaller and more stable effect on TEWL over time compared to shampoo 1, as shown in Figure 2. Statistical analysis did not show any significant changes, as *p*-values were 0.94 and 0.60 for shampoo 1 and shampoo 2, respectively. In both cases, the non-parametric Kruskal–Wallis test was used, the results of which confirmed the absence of statistically significant differences between TEWL values at the analysed time points.

In summary, the use of both shampoos resulted in an increase in TEWL, especially 15 min after application, but these changes did not reach statistical significance.

#### 3.2.3. Effect on Skin pH Value

**Figure 3:**
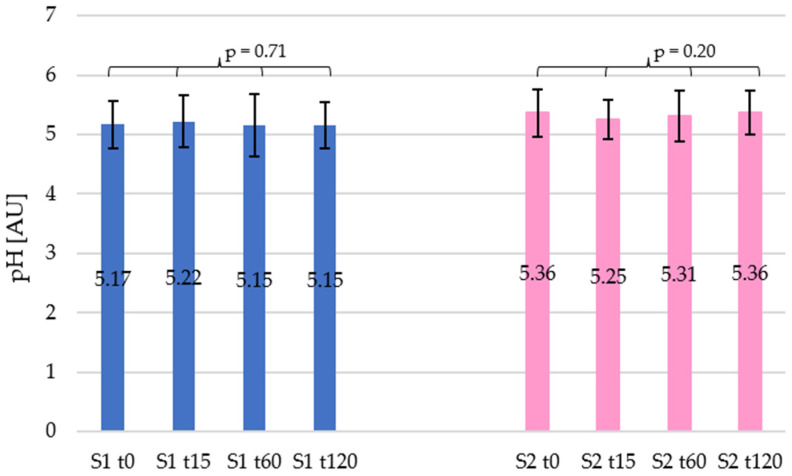
Changes in skin pH over time for the car shampoos tested. Blue and pink bars represent car shampoo 1 (S1) and car shampoo 2 (S2), respectively.

Performed instrumental and statistical tests confirmed that the skin pH values did not show any statistically significant differences after application of both tested shampoos (Figure 3). In the case of shampoo 1, after 15 min (t15), a slight increase in pH value of 0.91% was recorded, while at subsequent time points the difference values were 0.38% and 0.32% for t60 and t120, respectively. However, statistical analysis revealed that these changes were not significant (*p* = 0.71). For shampoo 2, a systematic decrease in pH was observed, but finally it returned to the initial value. At t15 and t60, a decrease in pH of 2.09% and 0.98%, respectively, was recorded, while after 120 min, the pH value was only 0.15% below the initial state. In this case, statistical analysis did not show any significant differences (*p* = 0.20), as well.

The results confirm that none of the analysed products had a significant effect on skin pH, which indicates their neutrality towards the natural hydrolipid barrier. This conclusion is further supported by the results of a one-way ANOVA analysis, which did not show any statistically significant changes in skin pH over time.

#### 3.2.4. Changes in Skin Topography

**Figure 4:**
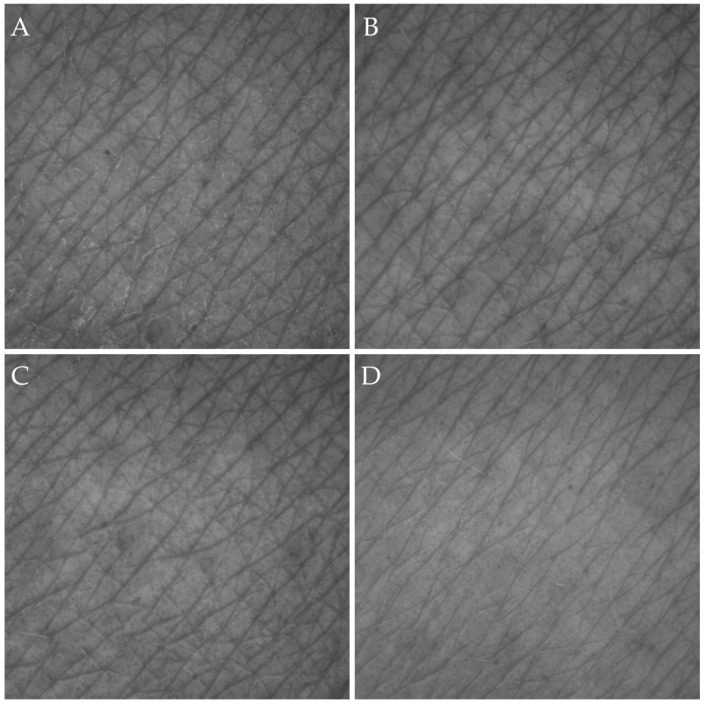
Example photos of the skin surface obtained for one of the participants using car shampoo 1 at the following measurement stages: (**A**)—before product application, (**B**)—15 min after application, (**C**)—60 min after application, and (**D**)—120 min after application.

**Figure 5:**
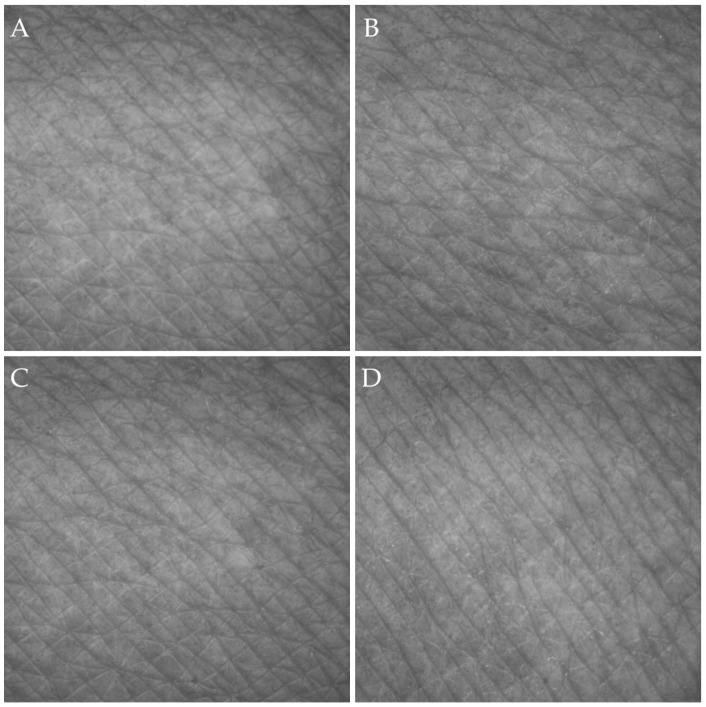
Example photos of the skin surface obtained for one of the participants using car shampoo 2 at the following measurement stages: (**A**)—before product application, (**B**)—15 min after application, (**C**)—60 min after application, and (**D**)—120 min after application.

**Figure 6:**
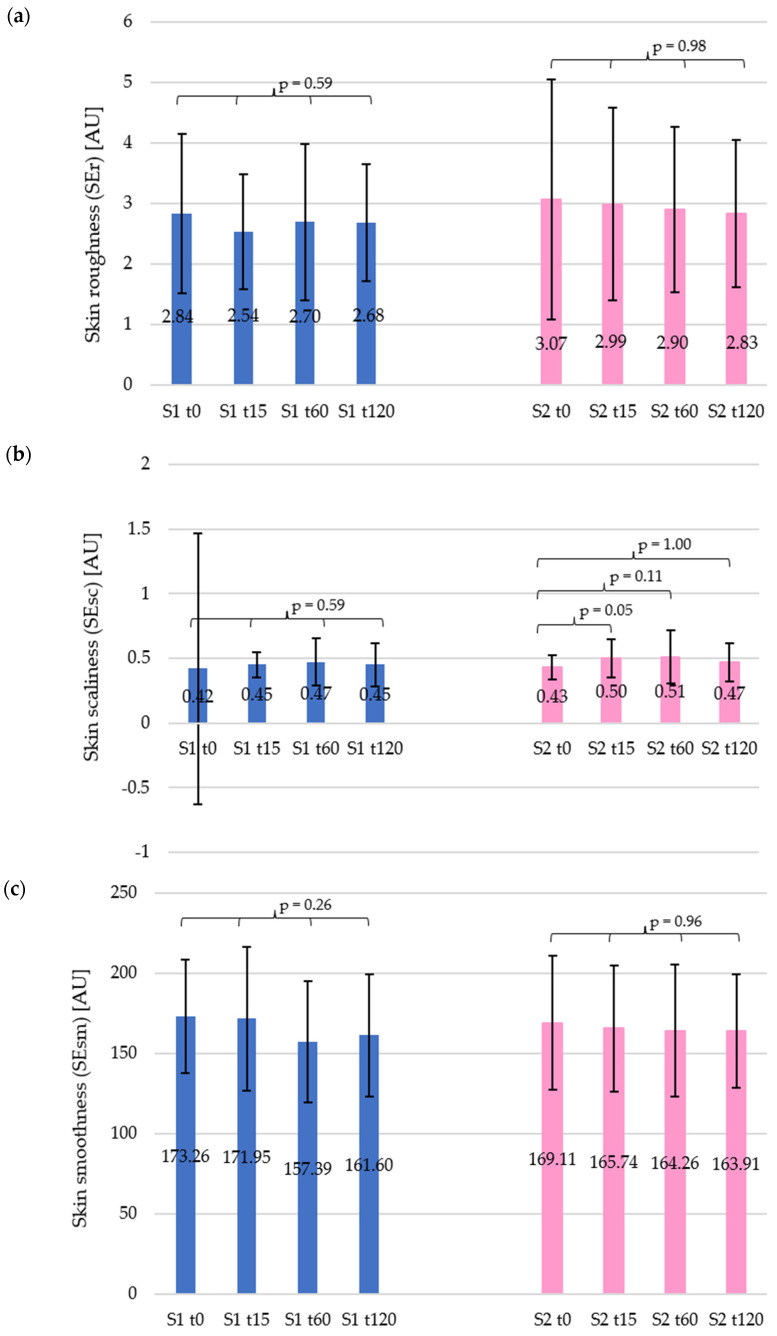
Changes in skin topography parameters over time for the car shampoos tested: (**a**) skin roughness (SEr), (**b**) skin scaliness (SEsc), (**c**) skin smoothness (SEsm). Blue and pink bars represent car shampoo 1 (S1) and car shampoo 2 (S2), respectively.

The following presents a detailed analysis of changes in selected skin topography parameters, namely roughness (SEr), scaliness (SEsc), and smoothness (SEsm). For each participant, a series of three photographs was taken for each forearm, at three fixed measurement points, at four designated time points (t0, t15, t60, and t120). Figure 4 and Figure 5 show examples of the images obtained for selected participants, illustrating the visual changes in the skin surface after the application of the products tested. Based on the images obtained, the average values of individual parameters were calculated, which were then used to analyse changes in their values over time (Figure 6).

In the case of both tested car shampoos, no negative impact of their use on the condition of the skin was observed (Figure 4 and Figure 5). Analysis of the photographs did not reveal any allergic reactions, irritation, redness, or signs of dryness. The results confirm that neither of the tested formulations causes any undesirable changes in the appearance and condition of the skin.

In terms of skin roughness, different skin reactions to both tested shampoos were observed (Figure 6a). For shampoo 1, a decrease in the parameter value of 10.60% was recorded in the initial phase of the study (t15), indicating some changes in skin topography. At t60, this difference decreased by 4.89%, while after 120 min (t120) an increase of 5.63% was observed. In the case of shampoo 2, the effect of the product in the first stage (t15) was much weaker, as the SEr parameter decreased by only 2.63%. However, after 120 min, this value shows a total decrease of 7.92% compared to t0. The data obtained suggest that both shampoo formulations had a mild effect on skin roughness. The statistical analysis performed using the Kruskal–Wallis test indicated no statistical significance for either of the products. For shampoo 1, a value of *p* = 0.59 was obtained, while for shampoo 2, it was *p* = 0.98.

In the case of skin scaliness measurements results (Figure 6b), different effects of the tested shampoos were observed. Statistical analysis using the Kruskal–Wallis test did not show any significant changes in the case of shampoo 1 (*p* = 0.59). However, a different effect was obtained for shampoo 2, for which statistical significance was found (*p* = 0.03). At t15, shampoo 1 showed an increase in the SEsc parameter value of 6.08%, while shampoo 2 showed a much stronger effect, reaching an increase of as much as 16.02% (*p* = 0.05). Sixty minutes after application of the product (t60), a further increase was observed in both cases. For shampoo 1, the change in SEsc parameter value more than doubled, reaching 12.27%, while for shampoo 2, the increase reached 17.95% (*p* = 0.11), relative to the t0 value. In the final measurement (t120), the changes in SEsc parameter values decreased for both products with very similar results, namely 7.54% for shampoo 1 and 7.87% for shampoo 2 (*p* = 1.00) compared to the initial value. These results indicate that shampoo 2 had a stronger effect on the skin exfoliation process in the initial phase of the study, but after 120 min, the differences between the products were negligible.

Analysis of skin smoothness parameters showed varied skin reactions to both shampoos tested (Figure 6c). For shampoo 1, the SEsm value at t15 remained almost unchanged from the initial level at t0, showing only a minimal decrease of 0.01%. However, subsequent measurements showed a clear decrease in value, namely at time t60, the decrease was 9.16%, while after 120 min (t120) it reached 6.73% relative to the initial value. Statistical analysis using the Kruskal–Wallis test did not show these changes to be significant (*p* = 0.26). Shampoo 2 had a smaller effect on skin smoothness, as initially (t15), a 2.00% decrease in the SEsm parameter value was recorded, and it did not change significantly in subsequent measurements: at t60, the decrease was 2.87% and at t120, it was 3.08%. Nevertheless, no statistical significance was found for this product either, as confirmed by a one-way ANOVA analysis (*p* = 0.96).

#### 3.2.5. Survey Results

**Figure 7:**
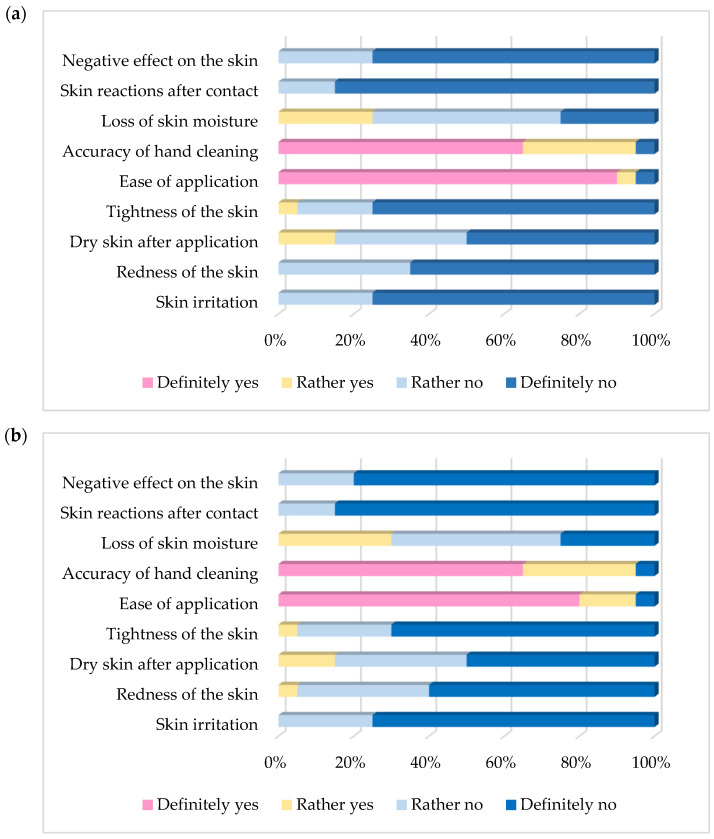
Results of the survey on the subjective assessment of the effect of tested car shampoos on the skin (**a**)—results for car shampoo 1, (**b**)—results for car shampoo 2.

The results of the surveys completed by 20 study participants, reflecting their subjective assessment of the products used, are presented in Figure 7a (shampoo 1) and Figure 7b (shampoo 2). As shown, both products exhibited very good skin tolerance, with no reports of adverse effects, allergic reactions, or irritation, with the majority of responses indicating “definitely not”. In the questionnaires, 5% of participants reported a sensation of skin tightness when using both products, and an additional 5% reported a possibility of slight skin redness when using shampoo 2. The vast majority of participants (95%) found the tested products very easy to apply and characterised by strong cleansing properties, with most responses indicating “definitely yes”. Overall, the results obtained for both formulations were highly similar, differing only marginally in certain aspects. In summary, both products were rated very positively in terms of their functionality and skin tolerance, with shampoo 1 receiving slightly higher comparative ratings.

## 4. Discussion

Both formulations of the car shampoos tested showed good skin tolerance. In dermatological tests, none of the 20 participants experienced skin reactions such as irritation, burning, or allergic responses, either 48 h or 72 h after application of the products.

During the application tests, both shampoos showed a similar course of moisture changes. Initially, a decrease in moisture was observed, which then returned to the initial value. The decrease was more pronounced for shampoo 2 (7.50% after 15 min) than for shampoo 1 (4.68% after 15 min). The values of difference, referenced to t0, increased successively until finally reaching 0.60% for shampoo 1 and 1.41% for shampoo 2 after 120 min. The temporary decrease in skin hydration observed within the first 15 min after application, particularly for shampoo 2, followed by a gradual recovery to baseline levels, is consistent with typical short-term desorption phenomena induced by surfactant-based cleansing systems. Similar transient reductions in corneometric hydration were reported by Huygen et al., who demonstrated that formulations containing mild surfactants initially remove a portion of the stratum corneum water, which is then restored through physiological equilibration [32].

Correspondingly, for both formulations, an initial increase in TEWL was observed, followed by a decrease over time. Shampoo 1 had a greater effect on TEWL, with an increase of 14.34% after 15 min, while shampoo 2 contributed to an increase of 7.27% during the same time. The increase in TEWL values observed after 15 min can be interpreted as a short-term barrier disturbance resulting from surfactant action, as already observed by Huygen et al. [32]. However, the subsequent normalisation of TEWL indicates the absence of cumulative damage. These results are in agreement with the findings of Lemery et al., who showed that mild cleansers may transiently elevate TEWL without long-term impairment of barrier integrity. This is connected with increasing the skin polarity without any extraction of the skin lipids and only their reorganisation, as previously observed for mild surfactants [40].

Both shampoos maintained the skin surface pH within the physiological range (4.5–5.5) throughout the study. No statistically significant deviations were recorded, confirming the neutrality of the formulations toward the acid mantle of the skin. These findings correspond to the observations of Lukić et al., who emphasised that pH-neutral or slightly acidic cleansing formulations better preserve the resident microflora and epidermal enzyme activity [41]. In contrast, alkaline products have been associated with prolonged pH elevation and barrier impairment. The very small pH fluctuations (<2% after 15 min) observed during our study demonstrate the appropriate buffering capacity of the tested formulations. For the other measurements, the differences did not exceed 1%, indicating that the products are neutral concerning the natural hydrolipid coat [42,43].

Parameters describing skin topography, i.e., roughness (SEr), scaliness (SEsc), and smoothness (SEsm), showed no statistically significant changes over time, which further substantiates the mildness of the tested products. The temporary reduction in roughness observed for shampoo 1 (−10.6% after 15 min) may indicate the removal of surface impurities and sebum residues, which was expected for the tested type of product. Comparable effects were noted by Lee et al., who reported short-term changes in skin microrelief after controlled cleansing procedures due to the removal of superficial corneocytes [44].

Shampoo 2 had a stronger effect on scaliness in the initial phase (+16.02%) compared to shampoo 1 (+6.08%). In the subsequent stages, both products caused a further increase in this parameter, that is, 12.27% for shampoo 1 and 17.95% for shampoo 2. However, 120 min after application, the changes in SEsc values, compared to the initial value at t0, were similar for both formulations, namely +7.54% and +7.87% for shampoo 1 and shampoo 2, respectively. Thus, it can be concluded that a slight but statistically significant increase in the scaliness parameter for shampoo 2 (*p* = 0.03) suggests enhanced desquamation activity, which might result from differences in surfactant structure or inclusion of mild polishing agents. Nonetheless, the recovery of this parameter after 120 min indicates that exfoliation remained within the physiological range. This reversible effect is consistent with enzymatic control of desquamation and cleanser-induced, transient modulation of corneodesmosome proteolysis [45,46] and with studies showing measurable but reversible changes in skin surface roughness after cleansing under mild conditions [44].

In the case of skin smoothness (SEsm), shampoo 1 did not initially cause a significant change (+0.01%), but this value then fell to −9.16% and gradually increased to −6.73% after 120 min. Shampoo 2 had a milder effect on the skin, with a decrease of 2.00% after 15 min, increasing to −2.87% after 60 min and −3.08% after 120 min. Taking into account the values after 60 min, it can be noticed that skin smoothness showed small, non-significant decreases at t60 (−9.2% for shampoo 1 and −3.1% for shampoo 2) that trended back toward baseline by 120 min. Transient changes in skin microrelief/roughness after mild cleansing have been documented in controlled in vivo studies using 3D topography methods [44,47]. Moreover, evidence on cleanser mildness indicates that systems combining anionic surfactants with amphoteric or non-ionic co-surfactants mitigate barrier perturbation and irritation versus purely anionic systems [43,48]. This aligns with the lack of statistical significance and the return of SEsm values toward baseline observed during our study.

Moreover, in the surveys performed during our study, 95% of participants found both products easy to apply and effective at cleaning. None of the participants reported any negative effects on the skin, allergic reactions, or irritation. In the case of shampoo 2, 2.5% of participants noticed slight reddening of the skin, which was temporary. It seems that such irritation can be caused by the higher content of Caprylyl/Capryl Glucoside [49].

The development of car shampoo concentrates that combine high cleaning efficiency with favourable safety and environmental profiles remains a significant challenge in car care products. In this context, the present work focused on two proprietary custom-developed formulations designed to exhibit excellent cleaning and foaming properties while ensuring safety for both the environment and the user’s skin. A key distinguishing feature of the proposed formulations is the exclusive use of highly biodegradable raw materials, including fragrances and colourants. This approach remains relatively uncommon among commercially available products in this category.

Commercial car shampoos are most often based on ionic surfactants such as SLES and SLS, which have been reported to negatively affect the skin barrier, potentially leading to irritation, increased TEWL, and reduced skin hydration [50,51]. In contrast, the developed formulations employ similar blends of biosurfactants, which differ primarily in the percentage composition of the individual components, namely Caprylyl and Capryl Glucoside. These formulations demonstrated the best cleaning and foaming properties, as reported in our previous studies [14]. Such performance characteristics are particularly important for this type of household product.

In conclusion, the results obtained confirm that both tested formulations are safe for dermal contact, as no significant alterations in fundamental skin parameters were observed. The products demonstrated very good skin compatibility, with only minor and transient variations in skin parameters such as hydration, transepidermal water loss, pH, and surface topography. These findings, together with the participants’ subjective assessments, indicate that the biobased ingredients incorporated into the formulations do not disrupt the physiological barrier functions of the skin, which is essential for ensuring the safety of vehicle cleaning products intended for frequent human contact.

## Data Availability

The original contributions presented in this study are included in the article/Supplementary Materials. Further inquiries can be directed to the corresponding authors.

## Supplementary Materials

The following supporting information can be downloaded at: https://www.mdpi.com/article/10.3390/ma19020269/s1, Table S1: Dermatological test results 72 h after application of the tested car shampoo formulations.

## Abbreviations

The following abbreviations are used in this manuscript:

TEWL	Transepidermal Water Loss
APGs	Alkyl Polyglycosides
FAEs	Fatty Alcohol Ethoxylates
NMF	Natural Moisturising Factor
SELS	Surface Evaluation of Living Skin
SEr	Skin Roughness
SEsm	Skin Smoothness
SEsc	Skin Scaliness
