# Supplementary material for: In Vivo Characterisation of Skin Response to Sustainable Car Cleaning Products
Source: Materials (Basel). 2026 Jan 9;19(2):269. doi: 10.3390/ma19020269 (PMC12843177; doi:10.3390/ma19020269)
Supplement: Supplementary file 1 [file materials-19-00269-s001.zip › materials-4036844-supplementary.pdf]

In Vivo Characterisation of Skin Response to Sustainable Car Cleaning Products

Bartosz Woźniak, Marta Marzec, Agata Wawrzyńczak, Izabela Nowak

Department of Applied Chemistry, Faculty of Chemistry, Adam Mickiewicz University in Poznań, Uniwersytetu Poznańskiego 8, 61-614 Poznań, Poland

Supplementary Materials

Table S1. Dermatological test results 72 hours after application of the tested car shampoo formulations.

Participant number	Skin type*	Test results after 48h**	Test results after 72h**
**U1**	D	0	0
**U2**	D	0	0
**U3**	N	0	0
**U4**	M	0	0
**U5**	N	0	0
**U6**	D	0	0
**U7**	N	0	0
**U8**	N	0	0
**U9**	N	0	0
**U10**	N	0	0
**U11**	M	0	0
**U12**	M	0	0
**U13**	D	0	0
**U14**	N	0	0
**U15**	N	0	0
**U16**	D	0	0
**U17**	D	0	0
**U18**	D	0	0
**U19**	D	0	0
**U20**	D	0	0

**Skin type: D – Dry, M – Mixed, N – Normal*

***Skin condition assessment: 0 – no reaction; 1 – slight erythema; 2 – erythema; 3 – erythema, papules; 4 – erythema, slight edema; 5 – erythema, infiltration, vesicles.*
